# Supplementary material for: Bovine tuberculosis in African buffalo (Syncerus caffer): Progression of pathology during infection
Source: PLoS Negl Trop Dis. 2022 Nov 11;16(11):e0010906. doi: 10.1371/journal.pntd.0010906 (PMC9683594; doi:10.1371/journal.pntd.0010906)
Supplement: Attachment — Submitted filename: Hilary Ann responses.docx [file pntd.0010906.s001.docx]

Methods

Reviewer #1: The authors tried to compare the distribution and progression of BTB lesion in buffalo with those of cattle. The progression of lesion in buffalo was investigated logitudinally. The objective of the study was clear. However, although it is possible to compare the types of tissues (lymph nodes) affected in the two species without running parallel experiments, it may not be possible to compare the progression of the infections between the two species without running parallell experimental infections under the same environmental conditions. Therefore, the authors cannot say much on the comparison of the progress of the infection in the two breeds.

We agree that running parallel experiments would be the correct way to compare progression, hopefully this article motivates such studies. We have tried to make clear in the discussion that these studies would be essential as we cannot state whether this is a species difference or a context difference – lines 349-351. “Ideally, parallel long term experiments comparing African buffalo to cattle studies could be performed, thus helping determine species versus context differences.”

Although the authors indicated that 38 buffalos were able to be investigated for pathological studies, the information given on the study subjects is not sufficient to understand detail of study subjects.

The methods state the description of the study subjects – see lines 135-150. Are there specific components the reviewer feels would be helpful. We are happy to add clarifications.

“This study utilized data from a previously conducted longitudinal study performed from 2008 to 2012. A free-ranging population of African buffalo in Kruger National Park, South Africa, was followed to investigate the consequences of anthelmintic treatment on BTB dynamics. Animals in the study were captured approximately every 180 days, over a 4-year period . As part of the initial study, animals were separated into an experimental group that received a long-lasting anthelmintic, and a control group, which were untreated. At each subsequent capture, the buffalo cohort were tested for BTB using a whole-blood interferon gamma (IFNγ) assay (BOVIGAM, Prionics, Switzerland). This assay measures the difference in IFNy production of whole blood in response to incubation with mycobacterial antigens (PPD) . For each animal, 2-9 BTB tests were obtained, depending on the time that each animal participated in the study (up to four years, 9 captures). For an animal to be considered positive for BTB, at least two consecutively positive BTB tests were needed . Animals with inconclusive BTB test results were excluded from our analysis. Of those culled and necropsied, 38 animals had a known conversion date within 6 months (based on capture period), and fully completed pathological exams. For the SNP 3195 analysis, sample size included 36 buffalo, with 12 risk heterozygotes, 4 risk homozygotes, and 20 wildtype buffalo. For the SNP 2253 analysis, sample size included 36 buffalo with 11 risk heterozygotes, 1 risk homozygotes, and 24 wildtype buffalo.”

Regarding the hypothesis, the authors argue that buffalo can serve as model for the investigation of TB in cattle and in humans. This argument is questionable or may be limited to buffalo raising countries if it works.

We agree this would be relevant to countries where wild bovines such as buffalo exist with TB.

Regarding the sample size, although the sample size of bovines used for comparison with the lesions of the 38 buffalos has not been described in the manuscript, the 38 buffalos used for this study are sufficient to generate data on the distribution and progression of lesion of BTB.

The methods state the description of the study subjects – see lines 135-150. See exept above

However, the pathology examination procedures described in the method section were not supported with references.

Thank you for this comment. That was an oversight and the pathology procedures are now cited out on lines 169-177.

As far as statistical analysis is concerrned, with my limited knowldge of statistics, I felt that the authors used appropriate statistical analysis. Furthermore, the
authors obtained approval from the ethical and regulatory bodies in order to undertake this study.
Thank you for your positive comments.

Reviewer #2: The objectives are clearly stated although the hypothesis is not stated as such.
The study design is appropriate and the population clearly described.
The correct statistical analyses appear to have been used.
There are not ethical or regulatory concerns.

Thank you for your comments

Lines 160-161: Why was length of infection determined from the initial positive IGRA result and to time of cull and subsequent histopathological examination? Shouldn’t the length of infection been from positive IGRA to euthanasia? Why was histopathological examination used in this determination?

This has been clarified in the manuscript, line 147

Lines 169-170: mandibular, parotid, palatine tonsil and retropharyngeal lymph nodes are generally considered cranial lymph nodes of the head rather than respiratory lymph nodes as stated by the authors.

Thank you for this clarification; correction has been made. “The distribution of macroscopic BTB lesions in the lungs, respiratory lymph nodes (caudal mediastinal **and tracheobronchial, and cranial lymph nodes of the hea**d (mandibular, parotid, palatine tonsil, and retropharyngeal) were assessed at time of **necropsy (14).”**

Line 173: what was done with the tissues collected where gross lesions were not seen?

Tissues unaffected were utilized for consumption, as approved by South Africa legislature.

Lines 22-234: In assessing the invasion of various tissues why was bacteriological culture not used?

We agree, bacteriological culture would have improved the study and was originally part of the assessment. However, due to tissue shipping issues, bacteriology was not completed.

Lines 159-160: The authors don’t justify why the metrics of lesion distribution, level of necrosis and extent of lesions in the lungs were used as measures of disease severity.
Clarification has been added lines 160-162. . “**While there is currently no standardized metric for disease severity postmortem in BTB, severity of pathological lesions and progression to necrosis as well as gross lesion burden have been utilized in disease modeling to assess severity (20).”**

Results
Reviewer #1: The results describes only the progression and distribution of the pathology of BTB in buffalos. But there are no data on pathology of BTB in bovine. The tissues and lymph nodes affected were described. The progress of the lesion was influenced with time. The figures are of poor quality. Their visibility should be improved. The Tables do not qualify the standard of Tables of a scientific paper and hence should be modified.

The tables and figures have been modified. We agree, a parallel study with bovines would be ideal; we utilized several previously completed studies from laboratory bovine studies to assess bovine pathology

Reviewer #2: The results are generally clearly stated.
Lines 255-256: what is meant by “rapid growth in the lymph nodes”? As written, is sounds as if the size of the lymph node was measured or bacterial burdens within lymph nodes were assessed.

Thank you for pointing this out; clarification has been made

Conclusions
Reviewer #1: The authors seem to describe their own opinions rather than focusing of their findings. The authors did not include the limitation of the study in their manuscript. While they have forwared their opinions on how the result of this study can be used in the future and also described the the relevance of this study to the public health.
We have tried to focus on the findings on our results and findings, in the context of the study itself, while acknowledging more work will need to be done within this field in order to fully understand buffalo as a potential model in addition to cattle for bovine tuberculosis. We have acknowledged cattle being studied in parallel would strengthen our argument. Thank you for your insight.

Reviewer #2: The conclusions are generally supported by the results and the limitations of the study are discussed.

Thank you for your feedback.

Lines 291-305: The authors focus on the differences in dosages as a means of accelerated spread of disease in cattle compared to the buffalo of the present study. It should also be mentioned that the routes of infection used by the references cited were different (intranasal vs intratonsilar), which may also affect disease progression.

Thank you, this clarification has been added lines 311-314 “**Additionally, the route of administration in cattle experiments differ, and affect the speed of progression. Intratonsilar tends to lead to a more rapid initial disease spread, while intranasal and intratracheal lead to a slower progression, and are thought to resemble a natural infectious process more closely** (**24**)**.”**

Other

Reviewer #1: The manuscript requires editorial work. The order of citations in the body of the manuscript should be corrected. For example, reference 6 should appear before reference 3. The use abbrevations should follow the usual way of using abbrevation. The quality and visibility of the figures should be improved. The Tables should fulfill the standard of Tables of a scientific paper.

I agree, and the requested edits have been made


Summary and General Comments
Use  this section to provide overall comments, discuss strengths/weaknesses of the study, novelty, significance, general execution and scholarship. You may also include additional comments for the author, including concerns about dual publication, research ethics, or publication ethics. If requesting major revision, please articulate the new experiments that are needed.

Reviewer #1: The study was conducted on the distribution and progression of BTB in buffalos. The data were generated from 38 buffalos. The authors try to compare the lesions in buffalo with the lesions in bovine. According to the authors the distribtions of the lesions in the two species are similar. Furthermore, the authors claim that the progression of lesion is faster in bovine than in buffalo although their study could not address this question. In summary, the progression and distribution of lesion of BTB in buffalo can be published.
Thank you very much for your detailed review. Your feedback was helpful and your expertise is appreciated.
Reviewer #2: This manuscript describes a 4-year project to examine the progression of Mycobacterium bovis infection in African buffalo. The authors monitor infection status through semiannual interferon gamma release assays (IGRA), using 2 positive sequential IGRAs as the rough start date of infection. Thorough postmortem and microscopic examinations were done to evaluate the severity and extent of disease. Additionally, the authors compared their results on disease severity to the presence of 2 different SNPS associated with disease progression.

The manuscript is generally well written and represents a great amount of work with a difficult species with which to work. The authors compare and contrast disease progression in African buffalo to published results from experimental infection studies in cattle, recognizing that the African buffalo are exposed to a broad range of environmental challenges not present in experimental settings.  Moreover, most experimental challenge studies in cattle do not last as long as the present study in buffalo due to the high cost of biocontainment.

Thank you very much for you time and your review.
